# Time-related association between fluid balance and mortality in sepsis patients: interaction between fluid balance and haemodynamics

**DOI:** 10.1038/s41598-018-28781-4

**Published:** 2018-07-10

**Authors:** Yanfei Shen, Weizhe Ru, Xinmei Huang, Weimin Zhang

**Affiliations:** 1 Department of Intensive Care Unit, Zhejiang Hospital, 12# Lingyin Road, Hangzhou, Zhejiang 322100 P.R. China; 2 Department of Oncology, Cixi People’s Hospital, No. 999, Nanerhuan East Road, Hushan Street, Cixi, Zhejiang 322100 P.R. China; 3 Department of Otolaryngology, Jinhua TCM hospital, No. 439, Shuangxi West Road, Jinhua, Zhejiang 322100 P.R. China

## Abstract

This study aimed to investigate the time-related association between cumulative fluid balance (FB) and mortality. Data were extracted from the Medical Information Mart for Intensive Care (MIMIC) III. FB data on 8584 patients at the first (FB-fir24hr) and second (FB-sec24hr) 24 hours after intensive care unit admission were analysed. Compared to the combination of FB-fir24hr ≤ 0 and FB-sec24 hr ≤ 0, the combination of FB-fir24hr > 0 and FB-sec24hr ≤ 0 had significantly higher FB, with an insignificant odds ratio (OR) for mortality. However, the mortality ORs of two other combinations (FB-fir24hr ≤ 0 and FB-sec24hr > 0; FB-fir24hr > 0 and FB-sec24hr > 0) were significantly high. Furthermore, multivariable logistic analysis showed a significant stepwise increase ORs for mortality with increasing FB-sec24hr quartiles, with no significant increase in FB-fir24hr quartiles aside from quartile 4. In patients with negative FB, a stepwise decrease in mortality ORs with increasing FB-sec24hr quartiles was found with no significant difference in FB-fir24hr quartiles. In conclusion, the positive FB during the second but not the first 24 hours was associated with increased mortality in sepsis. Achieving more negative FB was associated with decreased mortality only in the second 24 hours.

## Introduction

Fluid management is critically important in the initial stages of sepsis resuscitation. Since the 2001 landmark study by Rivers *et al*. reporting early goal-directed therapy^1^, several recommendations^2,3^ concerning fluid management have been suggested; these mainly consist of the use of an adequate and large volume of fluid resuscitation, especially during the first six hours after sepsis onset^4^.

As relative hypovolemia induced by vasodilatation disorder and capillary leak is common in patients with sepsis, appropriate fluid resuscitation is necessary to maintain adequate tissue perfusion. However, excess fluid administration can also lead to adverse outcomes, such as worsening respiratory function^5^, increased intra-abdominal pressure^6^, and coagulation disorder^7^. A post-hoc analysis in the Vasopressin and Septic Shock Trial (VASST) found that cumulative positive fluid balance (FB) over four days after admission was reported to be associated with higher mortality in septic shock^8^. Micek *et al*. also reported that the highest quartile of positive FB at eight days was an independent predictor of hospital mortality^9^. Consistent with these findings, another observational study^10^ found that the accumulated positive FB in the first 48, 72, and 96 hours was associated with higher mortality in sepsis. However, conclusions regarding the association between early positive FB and mortality are inconsistent. Several studies^11,12^ reported that positive FB within 24 hours after admission was not associated with increased death in sepsis, while other studies^8,13^ reported the opposite conclusion. There are several possible reasons for this. First, in patients with unstable haemodynamics, the detrimental effect of positive FB may be overwhelmed by the benefit of adequate fluid resuscitation, thus leading to an insignificant association. On the other hand, the true benefit of negative FB in this association may be overestimated, as the lowest FB quartile was commonly used as the reference quartile^8,11^. Whether there is a volume-related association between negative FB and mortality in sepsis remain unclear.

Therefore, this study had two main objectives: (1) To explore whether haemodynamic status played a role in the association between FB and mortality in sepsis, and (2) To explore whether achieving more negative FB would be beneficial in patients with sepsis. We speculated that a possible interaction between FB and haemodynamic status could lead to the disputed conclusions regarding the association between early positive FB and sepsis-related mortality.

## Results

### Comparisons of combinations

Data of 8584 patients with sepsis were included in this analysis, with an overall mortality rate of 21.8%. Comparisons of baseline characteristics between survivors and non-survivors are listed in Table 1. The mean age upon admission was 65.9 years, and 45.1% were male. Compared to Combination I (FB in the first 24 hours after ICU admission [FB-fir24hr] ≤ 0 and FB in the second 24 hours [FB-sec24hr] ≤ 0) (Table 2), Combination III (FB-fir24hr > 0 and FB-sec24hr ≤ 0) had significantly higher FB at 48 hours (FB-48hr) (−31.9 ± 19.9 vs. 17.7 ± 32.1, p < 0.001). However, the mortality rate between these two groups was quite similar (140/1040 (13.5%) vs. 226/1836 (12.3%), p = 0.373), and the adjusted odds ratio (OR) (Table 3) was insignificant in multivariable logistic regression analysis (OR, 0.86; 95% confidence interval [CI], 0.68–1.09, p = 0.206). The other two combinations (II: FB-fir24hr ≤ 0 and FB-sec24hr > 0; IV: FB-fir24hr > 0 and FB-sec24hr > 0) had significantly higher mortality (192/851 (22.5%) and 1320/4857 (27.1%), respectively) and the ORs were statistically significant (OR, 1.84; 95% CI, 1.44–2.35 and OR, 1.99; 95% CI, 1.64–2.42, respectively).

**Table 1:** Comparisons of baseline characteristics between survivors and non-survivors.

Variables	Overall (n = 8584)	Survivors (n = 6706)	Non-survivors (n = 1878)	p
Age (years)	65.9 ± 16.0	64.9 ± 16.3	69.4 ± 14.3	<0.001
Male [n (%)]	3878 (45.1)	3064 (45.6)	814 (43.3)	0.071
Weight (kg)	82.1 ± 26.0	82.7 ± 26.3	80.0 ± 24.8	<0.001
Emergency [n (%)]	7811 (90.9)	5998 (89.4)	1813 (96.5)	<0.001
Ethnicity				
White [n (%)]	6150 (71.6)	4843 (72.0)	1307 (69.5)	0.026
Black [n (%)]	662 (7.7)	546 (8.1)	116 (6.1)	0.005
Asian [n (%)]	201 (2.3)	159 (2.4)	42 (2.2)	0.773
ICU types				
MICU [n (%)]	4075 (48.6)	3030 (45.0)	1045 (55.6)	<0.001
SICU [n (%)]	998 (11.6)	883 (13.1)	115 (6.1)	<0.001
CCU [n (%)]	1147 (13.3)	865(12.8)	282 (15.0)	0.017
Infection sites				
Respiratory [n (%)]	5117 (59.6)	3858 (57.5)	1259 (67.0)	<0.001
Urinary [n (%)]	2780 (32.3)	2326 (34.7)	454 (24.1)	<0.001
Bloodstream [n (%)]	3038 (35.4)	2076 (30.8)	962 (51.2)	<0.001
Abdominal [n (%)]	1113 (12.9)	877 (13.0)	236 (12.5)	0.560
Intracranial [n (%)]	607 (7.1)	501 (7.6)	106 (5.6)	0.006
Laboratory indexes				
Maximum serum creatinine	2.19 ± 2.19	2.03 ± 2.21	2.76 ± 2.03	<0.001
Minimum serum haemoglobin	8.56 ± 1.72	8.63 ± 1.70	8.31 ± 1.76	<0.001
Maximum serum lactate	3.40 ± 3.17	2.90 ± 2.35	4.95 ± 4.58	<0.001
Maximum serum sodium	144.4 ± 5.2	144.2 ± 4.9	145.1 ± 6.1	0.001
Maximum white blood cell count	20.9 ± 12.5	20.3 ± 14.1	23.3 ± 15.3	<0.001
Clinical characteristics				
SOFA at ICU admission [median (IQR)]	5 (4–8)	5 (3–7)	6 (4–9)	<0.001
Fluid balance (ml/kg/48hr)	40.5 ± 56.7	34.9 ± 53.4	60.2 ± 63.2	<0.001
Fluid balance (ml/kg/first-24hr)	28.3 ± 40.6	26.1 ± 39.3	36.3 ± 44.1	<0.001
Fluid balance (ml/kg/second-24hr)	12.1 ± 28.3	8.8 ± 26.3	23.9 ± 31.8	<0.001
Fluid intake (ml/kg/48 hr)	93.91 ± 58.2	91.3 ± 55.6	103.5 ± 64.7	<0.001
Intravenous fluid intake (ml/kg/48 hr)	84.8 ± 58.6	81.8 ± 56.3	95.6 ± 64.7	<0.001
Output (ml/kg/48 hr)	53.5 ± 35.0	56.3 ± 34.9	43.3 ± 33.7	<0.001
Urine output (ml/kg/48 hr)	43.1 ± 30.2	45.9 ± 30.2	32.9 ± 28.1	<0.001
Haemodialysis	969 (11.2)	638 (9.5)	331 (17.6)	<0.001
Vasopressor-use	2513 (29.2)	1846 (27.5)	667 (35.5)	<0.001

Abbreviations: MICU, medical intensive care unit; SICU, surgical intensive care unit; CCU, coronary care unit; SOFA sequential organ failure assessment; ICU, intensive care unit; IQR, interquartile range.

**Table 2:** Characteristics and outcomes by four combinations of FB-fir24hr and FB-sec24hr.

Variables	FB-fir24hr ≤ 0 and FB-sec24hr ≤ 0 (n = 1040)	FB-fir24hr ≤ 0 and FB-sec24hr > 0 (n = 851)	FB-fir24hr > 0 and FB-sec24hr ≤ 0 (n = 1836)	FB-fir24hr > 0 and FB-sec24hr > 0 (n = 4857)	p
Hospital mortality [n (%)]	140 (13.5)	192 (22.5)^#^	226 (12.3)	1320 (27.1)^#^	<0.001
ICU LOS [median (IQR)]	4.1 (2.9–7.3)	5.7 (3.2–11.3)^#^	4.0 (2.8–7.6)	6.0 (3.3–11.5)^#^	<0.001
Hospital LOS [median (IQR)]	10.7 (4.8–17.0)	12.4 (7.7–20.8)^#^	10.8 (7.1–17.7)	13.2 (7.9–21.5)^#^	<0.001
SOFA on admission [median (IQR)]	4 (3–6)	4 (3–6)	5 (4–7)^#^	6 (4–8)^#^	<0.001
Fluid balance (ml/kg/48 hr)	−31.9 ± 19.9	1.9 ± 25.3^#^	17.7 ± 32.1^#^	71.3 ± 56.0^#^	<0.001
Fluid intake (ml/kg/48 hr)	42.1 ± 26.7	67.3 ± 40.6^#^	84.9 ± 47.8^#^	113.1 ± 59.9^#^	<0.001
Output (ml/kg/48 hr)	74.0 ± 34.7	65.4 ± 40.0^#^	67.1 ± 35.1^#^	49.8 ± 29.0^#^	<0.001
Urine output (ml/kg/48 hr)	64.2 ± 32.0	49.9 ± 31.8^#^	54.2 ± 30.9^#^	33.1 ± 24.7^#^	<0.001
Haemodialysis [n (%)]	84 (8.1)	77 (9.0)	156 (8.5)	652 (13.4)^#^	<0.001

Abbreviations: FB-fir24hr, fluid balance within the first 24 hours after ICU admission; FB-sec24 hr, fluid balance within the second 24 hours (25 to 48 hours) after ICU admission; ICU intensive care unit; LOS, length of stay; SOFA, sequential organ failure assessment; IQR, interquartile range.

Note: Comparisons between combination I and other combinations were made and ^#^ indicated p value < 0.001.

**Table 3:** Odds ratio of death according to four combinations of FB-fir24hr and FB-sec24hr in logistic regression.

Variables	Crude Odds ratio (95% CI)	p	Adjusted Odds ratio (95% CI)	p
FB-fir24hr ≤ 0 and FB-sec24hr ≤ 0	Ref.	—	Ref.	—
FB-fir24hr ≤ 0 and FB-sec24hr > 0	1.87 (1.47–2.38)	<0.001	1.84 (1.44–2.35)	<0.001
FB-fir24hr > 0 and FB-sec24hr ≤ 0	0.90 (0.71–1.13)	0.373	0.86 (0.68–1.09)	0.206
FB-fir24hr > 0 and FB-sec24hr > 0	2.39 (1.98–2.89)	<0.001	1.99 (1.64–2.42)	<0.001
SOFA on ICU admission			1.12 (1.10–1.14)	<0.001
Urinary infection			0.69 (0.61–0.78)	<0.001
Respiratory infection			1.46 (1.29–1.63)	<0.001
Haemodialysis			1.07 (0.87–1.31)	0.491
Maximum serum creatinine			1.06 (1.03–1.09)	<0.001
Serum sodium on ICU admission (<135 mmol/L)			1.35 (1.19–1.54)	<0.001
Platelet count on ICU admission (<150 *10^9/L)			1.20 (1.06–1.35)	0.003
Serum calcium on ICU admission (<8 mg/dl)			0.87 (0.78–0.90)	0.020

Abbreviations: FB-fir24hr, fluid balance within the first 24 hours after ICU admission; FB-sec24hr, fluid balance within the second 24 hours (25 to 48 hours) after ICU admission; ICU intensive care unit; SOFA, sequential organ failure assessment.

### Association between FB and mortality

#### Quartiles of all patients

Further multivariable logistic analysis (Table 4) showed a significant stepwise increase in mortality rate with increasing FB-sec24hr quartiles (from Q2 [OR, 1.56; 95% CI, 1.31–1.86] to Q4 [OR, 3.33; 95% CI, 2.82–3.92]); however, in FB-fir24hr quartiles (using Q1 as the reference quartile), no other significant difference occurred aside from Q4 (OR, 1.41; 95% CI, 1.20–1.65). This increasing trend of ORs became less significant in FB-48hr quartiles (from Q2 [OR, 1.20; 95% CI, 1.01–1.42] to Q4 [OR, 2.31; 95% CI, 1.88–2.59]).

**Table 4:** Adjusted odds ratio of mortality using fluid balance as design variables in logistic models.

Variables	Odds ratio (95% CI)	p	Odds ratio (95% CI)	p	Odds ratio (95% CI)	p
All patients						
Model 1	All FB-fir24hr (n = 8584)	All FB-sec24hr (n = 8584)	All FB-48hr (n = 8584)			
FB Q1	1	—	1	—	1	—
FB Q2	1.05 (0.89–1.23)	0.540	1.56 (1.31–1.86)	<0.001	1.20 (1.01–1.42)	0.035
FB Q3	1.11 (0.95–1.31)	0.164	1.94 (1.64–2.31)	<0.001	1.57 (1.33–1.85)	<0.001
FB Q4	1.41 (1.20–1.65)	<0.001	3.33 (2.82–3.92)	<0.001	2.31 (1.88–2.59)	<0.001
Patients with negative FB						
Model 1	Negative FB-fir24hr (n = 1882)	Negative FB-sec24hr (n = 2866)	Negative FB-48hr (n = 1950)			
FB Q1	1	—	1	—	1	—
FB Q2	0.86 (0.61–1.21)	0.391	0.97 (0.72–1.32)	0.891	0.87 (0.61–1.24)	0.459
FB Q3	0.88 (0.62–1.24)	0.480	0.62 (0.44–0.86)	0.005	0.74 (0.52–1.07)	0.115
FB Q4	0.77 (0.54–1.09)	0.145	0.59 (0.43–0.82)	0.002	0.53 (0.36–0.78)	<0.001

Abbreviation: FB-fir24hr, FB-sec24hr, and FB-48hr represented the fluid balance within the first 24 hours, the second 24 hours and the 48 hours after ICU admission, respectively.

Note: All three models were adjusted for infection site, disease severity score, haemodialysis, and laboratory factors (including serum creatinine, sodium, calcium and platelet count).

#### Quartiles of patients with negative FB

Patients who achieved negative FB during the three periods (the first and second 24 hours, and the entire 48 hours) were further divided into four quartiles (Table 4). Logistic regression analysis was used to explore the volume-related association. ORs of mortality decreased stepwise with increasing FB-sec24hr (high quartile indicating a more negative FB) (from Q2 [OR, 0.97; 95% CI, 0.72–1.32] to Q4 [OR, 0.59; 95% CI, 0.43–0.82]) and FB-48hr quartiles (from Q2 [OR, 0.87; 95% CI, 0.61–1.24] to Q4 [OR, 0.53; 95% CI, 0.36–0.78]), with no significant association in FB-fir24hr quartiles.

### Subgroup analysis of FB-fir24hr

As we speculated that haemodynamic status might have an interaction with FB, we performed a subgroup analysis according to the use of vasopressors within 24 hours after ICU admission. In patients without vasopressor-use, the mortality rate significantly increased stepwise with the increased quartile of FB-fir24hr (from Q2: OR, 1.05; 95%CI, 0.87–1.27 to Q4: OR, 1.32; 95%CI, 1.09–1.59) withinsignificant in patients with vasopressor-use aside from Q4 (see Supplementary Table S1).

### Fluid intake/output distribution

**Figure 1:**
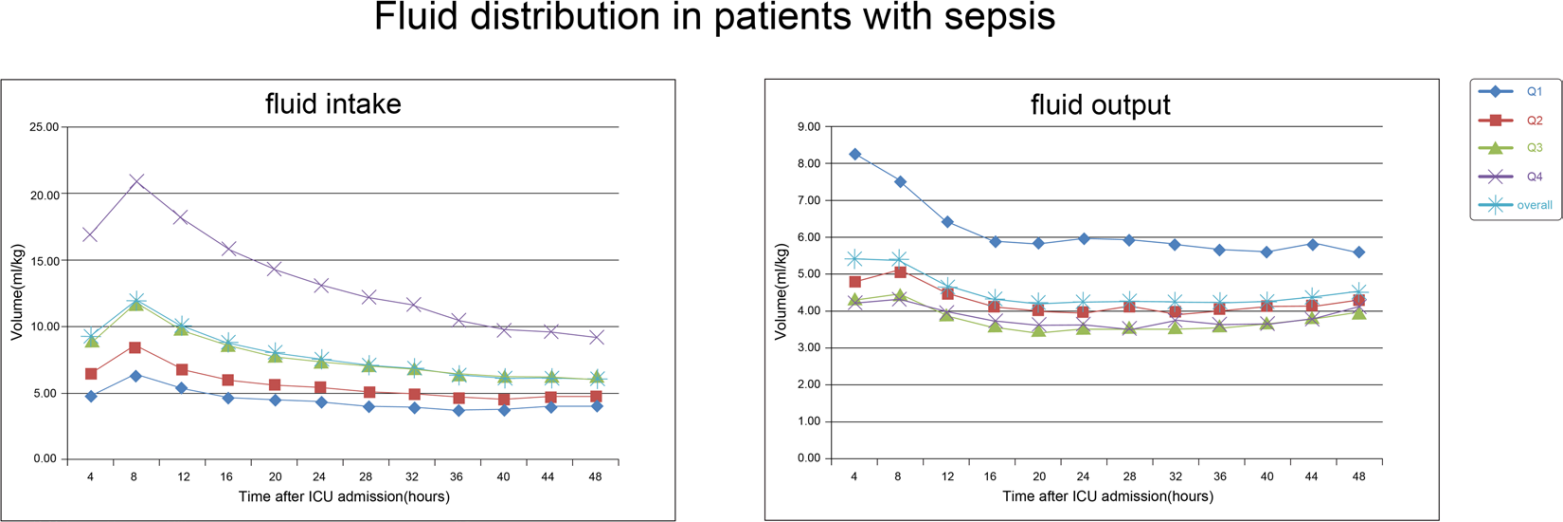
Volume distribution of fluid intake and output over the first 48 hours after ICU admission in patients with sepsis.

To explicitly show the fluid distribution in patients within each quartile, the amount of fluid intake/output is shown in four-hour intervals in Fig. 1. The fluid intake curve fluctuated during the first 24 hours and peaked within 4–8 hours, whereas the curve during the second 24 hours after admission was relatively smooth.

## Discussion

Our analysis, including 8584 patients with sepsis from a large online database (Medical Information Mart for Intensive Care III [MIMIC III]), revealed two key findings. First, daily FB in the second—but not the first—24 hours after ICU admission was positively associated with hospital mortality. Second, achieving more negative FB during this same time period (second but not first 24 hours) was associated with decreased mortality.

Associations between cumulative positive FB and adverse outcomes have been well established in patients with sepsis. We hypothesized that there was an interaction between early FB and haemodynamic status that contributed to the insignificant association between positive FB-fir24hr and mortality. Results from one large, multicentre, observational study indicated that the volume of positive FB was an independent risk factor for mortality^14^. The post-hoc analysis in VASST showed that cumulative positive FB over four days was associated with higher mortality in septic shock^8^, while Micek *et al*. also found the highest positive FB quartile at eight days was an independent predictor of hospital mortality^9^. Consistent with previous findings, we also found that high fluid accumulation at 48 hours was significantly associated with increased mortality. However, conclusions regarding the association between early FB and mortality are conflicting in reported studies. Boyd *et al*.^8^ and Sadaka *et al*.^13^ found that a more positive FB early in the resuscitation was associated with increased mortality in septic shock. However, in a multi-centre observational study, Sakr *et al*.^11^ reported otherwise, that early fluid resuscitation (within 24 hours after admission) was not associated with increased mortality in sepsis, and that it might even be associated with reduced mortality in patients that have septic shock for more than three days^12^. In the current study, we also found that early fluid accumulation (FB-fir24hr) was not associated with mortality rate. However, in the subgroup analysis, FB-fir24hr was still significantly associated with increased mortality in patients without vasopressor-use, while insignificant in the patients with vasopressor-use. Interestingly, a similar but insignificant pattern was also shown in Table 3 of the study by Sakr *et al*.^11^, as insignificant OR for mortality increased stepwise in the subgroup with no septic shock. As both the necessity of adequate fluid resuscitation and the detrimental effect of fluid accumulation have been well established, we speculated that the detrimental effect of positive FB accumulated during fluid resuscitation may be reduced or overwhelmed by the benefits of adequate fluid resuscitation. This speculation could also explain the inconsistent findings of significant^8,13^ or insignificant^12,15,16^ associations of early positive FB with increased sepsis-related mortality, as haemodynamic status was largely different between these cohorts. If this is the case, fluid accumulation during the resuscitation stage needs to be re-evaluated. Additionally, we noticed that, despite a significantly higher cumulative FB-48hr in Combination III than in Combination II (Table 2), the mortality in Combination III was significantly lower.

Furthermore, as the available data renders it impossible to identify patients who underwent fluid resuscitation, we presented the fluid intake/output distribution at the first and the second 24 hours (Fig. 1). Compared to the smooth curve at the second 24 hours, the fluid intake curve at the first 24 hours fluctuated and peaked within 4–8 hours after ICU admission, which, to a certain degree, implies that fluids during this interval may be administered therapeutically instead of by a protocol-driven programme. However, without additional information, we cannot definitively assume that fluctuations in the fluid intake curve indicate fluid resuscitation.

Based on the current evidence, conservative fluid administration has been widely adopted after achieving relatively stable haemodynamic status, to avoid further positive FB. However, the true benefit of negative FB in sepsis may be overestimated, as the lowest quartile of FB is commonly used as the reference quartile^8,11,13^. Balakumar *et al*.^17^ reported that among critically ill patients, negative FB was more associated with long-term mortality than euvolaemia. However, this conclusion became unstable^18^ in sensitivity analysis and the great heterogeneity within included patients may lead to potential bias. There are no known reports evaluating whether there is a volume-related association between negative FB and mortality in sepsis. In this study, we found that the volume in negative FB was associated with decreased hospital mortality only in the second, but not the first, 24 hours after ICU admission. Based on our hypothesis in this study, it is reasonable to speculate that negative FB is only “beneficial” after haemodynamic status is stable. However, the underlying mechanism of this association, and whether the amount of negative FB relied on previous cumulative FB, could not be inferred from our study and requires further investigation.

Based on our findings, we suggest that the interactive effect of fluid accumulation and fluid resuscitation should be re-considered when making the fluid management protocol decision in sepsis. In patients with poor haemodynamics, adequate fluid resuscitation is necessary, and fluid accumulation during this stage is unlikely to be associated with poor outcomes. However, subsequent fluid management is critical after the patient’s haemodynamic status becomes relatively stable. Additionally, it is beneficial in this stage to avoid further fluid accumulation or achieve more negative FB.

One advantage of the present study is the large sample size from the MIMIC III database. Additionally, the detailed data enabled us to present fluid distribution by hour, adjust for confounding factors, and perform subgroup analysis. However, information bias is still possible, and other unmeasured factors (such as haemodynamic targets and fluid administered before ICU admission) were unrecorded in the present study. A second limitation is that the database used in this study comprised data from patients admitted to an ICU between 2001 and 2012, but sepsis 3.0^4^ was used as inclusion criteria in the current study. Thirdly, septic shock is an important subgroup of sepsis; however, due to a lack of related information in this database, patients with septic shock could not be separated from patients with sepsis. Thus, we selected patients with a record of vasopressor-use as potential cases of septic shock. However, the related selection bias cannot be avoided; as an example, epinephrine may have been used for bradycardia rather than for septic shock. Finally, due to the retrospective nature of the study, an association between FB and mortality can only be inferred, and cannot be established as a causal relationship. Further studies focusing on the interaction between FB and fluid resuscitation are needed to verify this speculation.

In conclusion, we found in this large cohort study that positive FB-sec24hr was significantly associated with increased hospital mortality, while FB-fir24hr was not. However, in the subgroup without vasopressor-use, the association between FB-fir24hr and mortality became significant. The amount of negative FB during the second—but not the first—24 hours after ICU admission showed a stepwise association with decreased mortality in patients with sepsis.

## Methods

### Data sources

All of the data in this study were extracted from a large online international database, Medical Information Mart for Intensive Care III (MIMIC III), which is a single-centre database comprising information of patients admitted to the Beth Israel Deaconess Medical Center in Boston, Massachusetts^19,20^. This database comprises over 58 000 hospital admissions for 38 645 adults and 7875 neonates from 2001 to 2012, and the use of the database was approved by the institutional review boards of the Massachusetts Institute of Technology. The included patients’ information was anonymised, and thus the need for patients’ informed consent was waived for this study. All data were extracted by the corresponding author (certification number: 1564657), who passed the online training on protecting human subjects research participants.

### Inclusion and exclusion criteria

Adult patients meeting the criteria for sepsis were initially screened. Sepsis definition was adapted from the recommendations in Surviving Sepsis Campaign 2016^4^, defined as sequential organ failure assessment (SOFA) score ≥2 within 24 hours after ICU admission, accompanied by at least one infection site. To accurately identify patients with a diagnosis that suggested sepsis, we manually reviewed all International Classification of Diseases, Ninth Revision codes indicating the existence of infection. The following criteria were used to exclude patients from this analysis: (1) younger than 18 years, (2) spent less than 48 hours in the ICU, and/or (3) without data on fluid management. For patients who stayed in the ICU more than once, only the first ICU stay was used in this study.

### Grouping method

Cumulative FB was calculated as follows: (fluid intake - fluid output) in millilitre/body weight (ml/kg). Fluid intake indicated all fluids (including colloid, crystalloid, or blood products) administered to the patient within 48 hours after ICU admission. As the main purpose of this study was to investigate the time-related association, daily FB instead of cumulative FB was determined. Thus, patients were stratified into four categories according to the four combinations of daily FB-fir24hr and FB-sec24hr after ICU admission. The categories were: Combination I (FB-fir24hr ≤ 0 and FB-sec24hr ≤ 0), II (FB-fir24hr ≤ 0 and FB-sec24hr > 0), III (FB-fir24hr > 0 and FB-sec24hr ≤ 0), and IV (FB-fir24hr > 0 and FB-sec24hr > 0). Furthermore, all patients were further stratified into four quartiles. Logistic regression analysis was performed to explore the time-related association between FB and mortality. In all logistic models, quartile 1 represented the lowest FB and quartile 4 represented the highest FB. For negative FB, quartile 1 represented the least negative and quartile 4 represented the most negative FB. The primary endpoint was hospital mortality, defined as observed death during hospitalisation.

### Subgroup analysis

Subgroup analysis was performed to explore possible interactions between FB and haemodynamic status. All patients were divided into two subgroups according to the use of any vasopressor within 24 hours after ICU admission, including norepinephrine, dopamine, dobutamine, and epinephrine.

### Missing data management

Variables with missing data were common in the MIMIC III database. In this analysis, the percentages of most missing variables were less than 5%. Thus, we replaced missing variables with their mean/median value^21^.

### Statistical analysis

Continuous variables were expressed as mean ± standard deviation (SD) or median (interquartile range) as appropriate. Categorical data were expressed as proportions^22^. Variables, including demographic characteristics, infection site, disease severity score, and laboratory measures potentially associated with mortality or which had a p value < 0.20 in univariate analyses were included in the multivariate logistic regression analyses^18,23^. Multi-collinearity was tested using variance inflation factor (VIF) method, with VIF ≥ 5 indicating the existence of multi-collinearity. The goodness of fit was checked for all logistic regression models. The two-tailed test was used, and p < 0.05 was considered statistically significant. All statistical analyses were performed using the software STATA 11.2 (StataCorp LLC, College Station, TX, USA).

### Data availability

The data that support the findings of this study were extracted from the MIMIC III database, https://physionet.org/works/MIMICIIIClinicalDatabase/files/, by the corresponding author, who passed the online training. Thus, the data are only available from the corresponding author upon reasonable request and with permission of MIMIC III.

## Electronic supplementary material

Subgroup analysis
