# Supplementary material for: Time-related association between fluid balance and mortality in sepsis patients: interaction between fluid balance and haemodynamics
Source: Sci Rep. 2018 Jul 10;8:10390. doi: 10.1038/s41598-018-28781-4 (PMC6039532; doi:10.1038/s41598-018-28781-4)
Supplement: Supplementary file 1 — Subgroup analysis [file 41598_2018_28781_MOESM1_ESM.pdf]

Time-related association between fluid balance and mortality in sepsis patients: Interaction between fluid balance and haemodynamics

Yanfei Shen[1*], Weizhe Ru[2#], Xinmei Huang[3], Weimin Zhang[1]

[1] Department of Intensive Care Unit, Dongyang People's Hospital, No. 60, Wuning West Road, Dongyang, Zhejiang, 322100, P.R. China

[2] Department of Oncology, Cixi People's Hospital, No. 999, Nanerhuan East Road, Hushan Street, Cixi, Zhejiang, 322100, P.R. China

[3] Department of otolaryngological, Jinhua TCM hospital, No. 439, Shuangxi West Road, Jinhua, Zhejiang, 322100, P.R. China

[#] Author Weizhe Ru contributed equally to this study and is listed as co-author

* Corresponding author:

Yanfei Shen

Department of Intensive Care Unit, Dongyang People's Hospital,

No. 60, Wuning West Road, Dongyang, Zhejiang, 322100, P.R. China

Phone: +86-137-3890-3218

E-mail: snow.shen@hotmail.com

Table S1 Adjusted odds ratio of death using fluid balance within the first 24 hours as design variables in logistic models

Variables	Odds ratio (95% CI)	p	Odds ratio (95% CI)	p
**Model 1**	Without Vasopressor-use (n = 6071)		Vasopressor-use (n = 2513)	
**FB Q1**	1	-	1	–
**FB Q2**	1.05 (0.87 – 1.27)	0.567	0.92 (0.69 – 1.22)	0.564
**FB Q3**	1.24 (1.03 – 1.50)	0.022	1.15 (0.87 – 1.52)	0.302
**FB Q4**	1.32 (1.09 – 1.59)	0.003	1.69 (1.29 – 2.22)	< 0.001
**SOFA on ICU admission**	1.14 (1.11 – 1.17)	< 0.001	1.13 (1.09 – 1.17)	< 0.001
**Urinary infection**	0.72 (0.62 – 0.83)	< 0.001	0.61 (0.49 – 0.76)	< 0.001
**Respiratory infection**	1.54 (1.33 – 1.77)	< 0.001	1.46 (1.20 – 1.78)	< 0.001
**Haemodialysis**	1.26 (0.98 – 1.62)	0.066	0.84 (0.60 – 1.17)	< 0.001
**Maximum serum creatinine**	1.05 (1.01 – 1.09)	0.005	1.11 (1.05 – 1.17)	< 0.001
**Serum sodium on ICU admission (< 135 mmol/L)**	1.31 (1.12 – 1.54)	< 0.001	1.52 (1.22 – 1.90)	< 0.001
**Platelet count on ICU admission (< 150 * 10^9/L)**	1.18 (1.02 – 1.37)	0.025	1.04 (0.83 – 1.29)	0.706
**Serum calcium on ICU admission (< 8 mg/dl)**	0.84 (0.73 – 0.96)	0.011	1.04 (0.84 – 1.27)	0.698

Abbreviations: CI, confidence interval; FB-48hr, fluid balance within the 48 hours after ICU admission; FB-fir24hr, fluid balance within the first 24 hours after ICU admission; ICU, intensive care unit; FB-sec24hr fluid balance within the second 24 hours (25 to 48 hours) after ICU admission; SOFA, sequential organ failure assessment.
